# Comparison of Two Distraction Devices for Assessment of Passive Hip Laxity in Dogs

**DOI:** 10.3389/fvets.2020.00491

**Published:** 2020-08-19

**Authors:** Ana Santana, Sofia Alves-Pimenta, João Martins, Bruno Colaço, Mário Ginja

**Affiliations:** ^1^Faculty of Veterinary Medicine, Lusófona University, Lisbon, Portugal; ^2^CITAB—Centre for the Research and Technology of Agro-Environmental and Biological Sciences, University of Trás-os-Montes and Alto Douro, Vila Real, Portugal; ^3^Department of Animal Science, University of Trás-os-Montes and Alto Douro, Vila Real, Portugal; ^4^Department of Veterinary Science, University of Trás-os-Montes and Alto Douro, Vila Real, Portugal

**Keywords:** hip laxity, distraction index, reproducibility, PennHip, Dis-UTAD, dog

## Abstract

Canine hip dysplasia is the most common orthopedic developmental condition in the dog and early hip laxity is the main risk factor. The importance of hip laxity in young animals in the development of hip dysplasia is unanimously recognized among researchers and veterinarians due to its medical applicability in terms of disease control and prevention. In the market, there is some certified hip distractors to promote joint laxity. However, the clinical use of some of these distractors complies with a set of usage rules, that can limit its medical application. In this study was compared the technical quality of radiographs and hip distraction using a certified hip distractor (CertD) and Dis-UTAD in 104 dogs (208 joints). The mean pelvic tilting of 1.5 ± 1.6° and 1.5 ± 1.8° were similar when using the CertD and the Dis-UTAD distractors, respectively (*P* > 0.05). In the CertD sample, the mean hip distraction index (DI) was 0.46 ± 0.17 and in the Dis-UTAD 0.46 ± 0.16; the mean DI differences was 0.001 ± 0.045, resulting in a non-significant paired *t*-test (*P* = 0.65) and a significant intraclass correlation coefficient of 0.96, with the 95% lower limit confidence interval of 0.95 (*P* < 0.05). The statistical power analysis showed a very low distraction index difference effect size. The results suggest that the statistical reproducibility of CertD hip distraction by the Dis-UTAD and the DI mean differences of 0.001 might be considered without clinical importance. The Dis-UTAD might be considered adequate to promote dog hip laxity.

## Introduction

Canine hip dysplasia (CHD) is a complex, inherited, polygenic trait disease influenced by multiple environmental factors, which was first identified in dogs by Schnelle in 1935 (1–3). CHD is considered as one of the most common orthopedic developmental conditions in dogs that lead to a debilitating secondary hip osteoarthritis (4). Although the etiology of CHD is not completely understood, increased laxity of the hip joint is the most frequent early cause reported and usually results in secondary osteoarthritis (OA) (5). CHD is a challenging disease to prevent, diagnose, and manage. Clinical signs such as decreased activity, difficulty in rising, “bunny hopping” gait, hind limb lameness, and hip pain support the suspicion of the disease (6). The actual diagnosis is confirmed radiographically if characteristic signs are evident on standard hip extended view (HEV) in dogs over 1 year of age (4). There is not an adequate molecular diagnosis for hip dysplasia (3); therefore, radiographic diagnosis has been essential for the selection of breeding stock and is based on two main key features: the detection of signs of degenerative joint disease or the diagnosis of early hip joint laxity (HJL) (7, 8). Although HEV has been shown to be a valuable tool in evaluating the presence of OA, it can severely underestimate HJL because of the non-physiological tensioning of the pelvic muscles and twisting of the joint capsule (5). Distraction–stress radiography techniques are used to better estimate the degree of passive HJL through the calculation of distraction index (DI) (5, 9). In the hip distraction view, the femoral heads are displaced laterally by the use of a custom-designed device (distractor) placed between the legs that acts as a fulcrum on the femur at the level of the ventral aspect of the pelvis (5). The DI is obtained by dividing the lateral femoral head displacement by its radius; a DI of 0 represents absolute joint congruity and a DI of 1 represents complete joint luxation (5). The hip joint distractors PennHIP (5, 7) and “FSA—Fondazione Salute Animale” (9) have been used in published works to obtain hip distraction views.

The purpose of the present study was to compare the technical quality of radiographs and hip distraction using the CertD and the Dis-UTAD, a hip distractor developed at the University of Trás os Montes e Alto Douro (UTAD).

## Materials and Methods

### Animals

In this prospective study, 104 dogs (58 females and 46 males) from five different breeds (68 Estrela Mountain dogs, 12 Transmontano Cattle Dog, 12 Portuguese Pointer Dog, 11 Rafeiro do Alentejo, and one Barbado da Terceira) were presented at the Veterinary Teaching Hospitals of University Lusófona de Humanidades e Tecnologias and University of Trás-os-Montes and Alto Douro between the years of 2014 and 2019 and screened for passive hip laxity. The recorded data included breed, age at the time of radiography, sex, and body weight. The inclusion criteria were that dogs had to be from Portuguese breeds, between 4 and 12 months of age at the time of the exam, and presenting normal musculoskeletal development upon clinical examination. The minimum sample size was estimated using a *t*-test table and selecting a statistical significance of 0.05, a medium variable effect size of 0.4, and a statistical power of 0.8, which indicated a sample of 99 observations (10).

All the radiographic examinations were performed with the dog owner's consent and all the animal procedures were undertaken as part of the work described in this study, performed in compliance with the regulations of our institutions (no. 1044-e-DCV-2018) and in accordance with the Portuguese and European regulations for animal use and care (European Directive 2010/63/EU and National Decree–Law 113/2013).

### Radiographic Procedure

**Figure 1:**
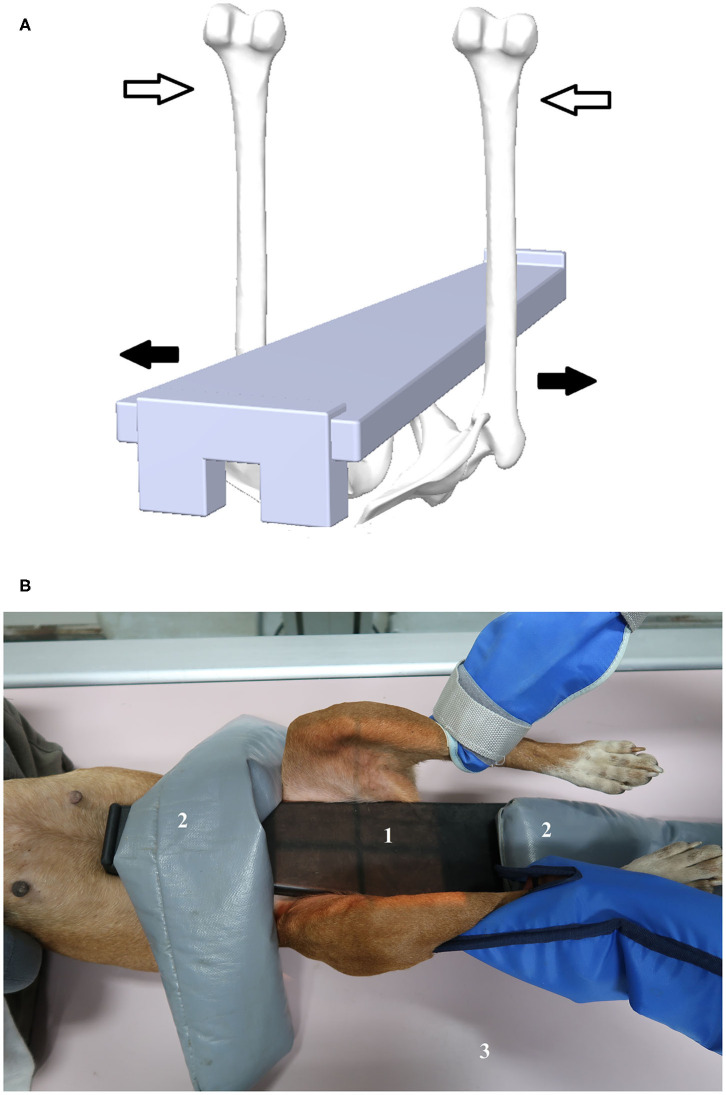
The Dis-UTAD is a modified Vezzoni distension device. **(A)** Illustration outlining the rear view of the dog and the Dis-UTAD. The open arrows represent the medial force exerted by the examiner, pushing the femurs against the distractor, and the full arrows represent the resulting hip distraction force. **(B)** Dis-UTAD positioned on the animal, fixed by the support of two sandbags. 1, Dis-UTAD; 2, sandbags; 3, X-ray table.

**Figure 2:**
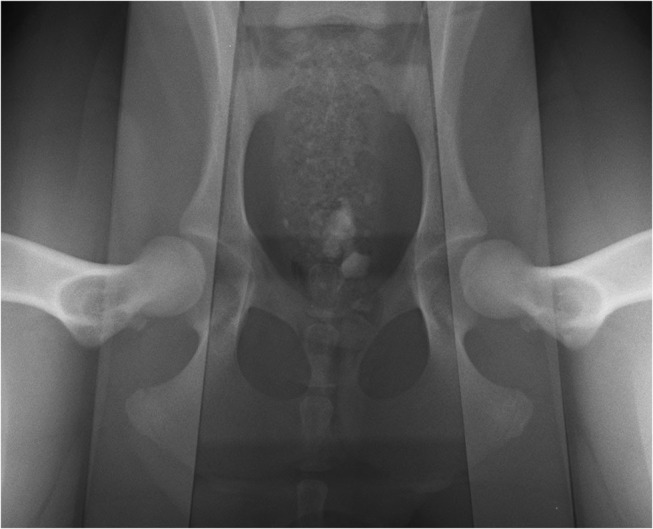
Hip distraction view using the Dis-UTAD distractor. The pelvis and the distractor are centered and symmetrical, and the more pronounced lateral band opacity of the distractor overlaps the femoral heads.

The radiographs were performed with the dogs under deep sedation using medetomidine (Domitor: Orion Corporation, Espoo, Finland) and butorphanol (Torbugesic Injectable: Fort Dodge Veterinaria, Girona, Spain) administered intravenously. The sedation was reversed with atipamezole hydrochloride (Antisedan: Orion Corporation, Espoo, Finland) intramuscularly. Radiographs were obtained in the same sequence with the dogs in dorsal recumbency on the X-ray table: first the HEV and then two distraction views with the distractor device placed between the hind limbs to promote passive hip laxity, first using the CertD and followed by the second distraction view using the Dis-UTAD. The Dis-UTAD is a modified Vezzoni distension device with an isosceles trapezoid shape (9); it has an external rubber component and a polyethylene plate in the interior that gives it longitudinal flexibility and transverse stiffness (11). With the dog in dorsal recumbency, hip distraction was achieved in a similar way to the PennHIP and Vezzoni techniques (5, 9). Both femurs were adducted by the examiner symmetrically in a neutral position (+/−10 degrees) against the distractor, fixed on the animal with the support of two cylindrical sandbags weighing ~4 kg each, one at the front and one at the distractor's back (Figure 1). On the distraction radiograph, the pelvis and the distractor should appear centered and symmetrical, and the more pronounced lateral band opacity of the distractor overlaps the femoral heads (Figure 2). The hip distractor fixation with sandbags was already described previously to avoid the exposure of the examiner's assistant to ionizing radiation (12). The radiographs were taken by veterinarians with experience in hip stress views (AS and MG).

### Positioning Evaluation and Hip Laxity Measurement

Technical radiographic positioning analysis was performed by estimating the grade of pelvis tilting (*y*) and measuring the asymmetry of the obturator foramen width (OFW) (*x*), *y* = 1.6*x* – 0.9 (13). For the hip laxity measurements, a dedicated semiautomatic software was used as previously described (14). The DI was calculated by dividing the distance between the centers of the femoral head and the acetabulum by the radius of the femoral head, as described previously (5). Both measurements of pelvic tilting and DI were performed in two independent sessions by JM and AS, respectively.

### Statistical Analysis

Statistical analysis was performed using the computer software SPSS (SPSS Statistics for Windows Version 23.0: IBM Corp., Armonk, NY, USA). The data analysis was performed on joints individually. The paired *t*-test and the intraclass correlation coefficient (ICC) were used in comparing the pelvic tilting and the DI of both hip distractors in order to evaluate Dis-UTAD's reproducibility (15, 16). A value of *p* < 0.05 was considered to be statistically significant. The null hypothesis was that the mean difference between paired observations was zero (10). The size effect and the statistical power were estimated to evaluate the ability of our sample to detect variable differences on each distractor set (10).

## Results

**Figure 3:**
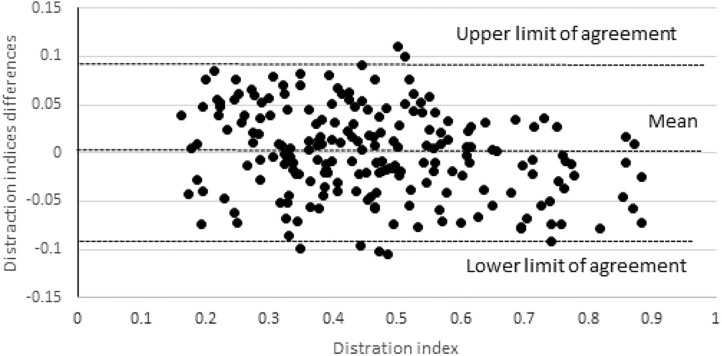
Differences between the distraction index obtained in distraction radiographs using the certified distractor and the Dis-UTAD. The horizontal lines represent the mean of the differences (0.001) and the upper and the lower 95% limits of agreement, ~0.09 and -0.09, respectively.

The dogs' age ranged from 4 to 11 months (mean ± standard deviation, 6.0 ± 2.1 months), and body weight ranged from 13.5 to 54 kg (mean, 24.7 ± 8.6 kg). In the CertD sample, the pelvic tilting ranged from 0 to 6.5° (mean, 1.5 ± 1.6°) and the DI ranged from 0.16 to 0.88 (mean, 0.46 ± 0.17). In radiographs obtained with the Dis-UTAD, pelvic tilting ranged from 0 to 6.3° (mean, 1.5 ± 1.8°) and the DI ranged from 0.12 to 0.88 (mean 0.46 ± 0.16). Comparing CertD and Dis-UTAD, for pelvic tilting the mean of the difference was 0.04 ± 1.9° and for DI it was 0.001 ± 0.045°, and the paired *t*-test was not statistically significant in both evaluations, being *P* = 0.84 and 0.65, respectively (Table 1 and Figure 3). The ICC between both DI samples for single measures was 0.96 (95% confidence interval, 0.95–0.97).

**Table 1:** Paired variable differences between CertD and Dis-UTAD.

**Variable**	***N***	**Paired differences**						***r***	**Effect size**	**Power**
		**Mean**	**SD**	**SEM**	**95% CI**		***P***			
					**Lower**	**Upper**				
PT (^°^)	104	−0.04	1.87	0.18	−0.4	0.33	>0.05	0.4	0.02	0.05
DI	208	0.001	0.045	0.003	−0.005	0.008	>0.05	0.96	0.03	0.07

° *, degrees; CI, confidence interval; DI, distraction index; N, number; P, statistical significance; PT, pelvic tilting; r, Pearson correlation; SEM, standard error of the mean; SD, standard deviation*.

## Discussion

Hip joint laxity is the main risk factor for the development of degenerative joint disease in dogs and is associated with high heritability (3, 8). Therefore, the use of DI breeding selection is highly recommended in the control programs of CHD (4). The clinical achievement of the distraction view requires some specific training and PennHIP has free online courses available. However, the clinical use of some hip distractors complies with a set of imposed rules that can limit its clinical usage (9, 17). The Dis-UTAD was developed to overcome some restrictions in hip distractor availability and intended, for interested veterinarians, as an adequate alternative in the assessment of dog's hip joint laxity. However, like the usage of other hip distractors, a previous practical training is recommended to perform adequate hip distraction views as well as for the distraction index measurement (14).

In this work, the OFW was used to evaluate the grade of pelvis tilting and not the iliac horizontal diameter as recommended in a previous work that used the HEV (13) because, in some radiographs of our sample, the X-ray collimation did not allow the observation of all sacroiliac joints. Nonetheless, this study showed also a good correlation between OFW and pelvic tilting (13). Our work shows that the degree of pelvic tilting using Dis-UTAD is similar to the one that we obtain by using the CertD. The mean degree of pelvic tilting in our samples (1.5°) was slightly higher than those in other works where conventional hip ventrodorsal view was used (13, 18). There are no previous published distraction hip studies where the degree of pelvic rotation has been evaluated. Theoretically, the tilting of the pelvis should not have much interference in DI measurement since this variable results from two spherical anatomical structures in a similar dorsal dog anatomical plane and relatively close to the center of the X-ray beam. Radiographic spatial distortion is especially important in the periphery of the X-ray beam and when the reference structures are at different distances from the radiographic film (19).

The non-significant *t*-test and the ICC of 0.96 with a lower limit of the 95% CI ≥ 0.75 indicate that there is no bias, a strong association between the hip distraction promoted by both distractors and statistical reproducibility and interchangeability (16). However, the low statistical test power does not allow us to reject the false null hypothesis (10). As the mean DI difference effect size is very low (0.07), we will need a sample of more than 1,500 animals to obtain enough statistical power (0.80) to demonstrate that the DI differences are not due to the Dis-UTAD distractor (10). However, when the mean variable differences are exceedingly small, they can be considered without medical importance (20, 21). There is no statistical test powerful enough to detect variable differences between samples with a mean of 0 since an infinite sample would be needed for comparison (21). The DI measurement differences included also examiner and scoring errors (22), which are difficult to differentiate. A similar reproducibility of the PennHIP method results was obtained in a recent study using the Vezzoni Modified Badertscher distractor (9). The longitudinal flexibility of the Dis-UTAD allows good adaptation to the dog's body and the sandbags stabilize it adequately, resisting better to the examiner's medial force on the hind limbs. Distractor stabilization is also needed in the PennHIP distractor using sandbags (12) or the help of an assistant (5), which we do not recommend because it increases human exposure to ionizing radiation.

The higher lateral band opacity of the distractor, overlying the femoral heads, gives the examiner a good idea of the symmetry of positioning and distraction level (Figure 2). It is recommended to repeat the examination when this does not happen. The rear Dis-UTAD thicker component with a central hole allows table support, accommodates the tail of the dog, and allows a more horizontal use of the distractor, characteristics that facilitate the hip distraction process. The heterogeneity of our sample (animals of about 10–50 kg) also shows that Dis-UTAD has the ability to promote adequate hip distraction in small and large dogs using its cranial or caudal part in distraction, respectively. Nonetheless, the small number of breeds can be pointed out as a limitation of this study. The DI ICC in this study (0.96) was higher than the within- (0.94) and between-examiner (0.91) DI repeatability of previous studies using the PennHIP distractor (17) and similar to other studies that evaluate the reproducibility of PennHIP DI measurements (22). These facts may be associated with the examiner's expertise and the reliability of the DI measurement method or may be related to the size or the type of the sample used. The dedicated semiautomatic software used in hip laxity measurements was already used in a previous work, which proved to be effective (14).

This and other recent studies show that joint laxity can be reliably quantified with the use of different distractors and methods (9), and there are scientific and technical conditions for extending their use in breeding selection and for preventive CHD management purposes. The recognition of the importance of hip laxity in young animals in the development of hip dysplasia is unanimous among researchers and veterinarians (5, 7, 9, 23, 24), so its medical applicability in terms of disease control and prevention should be promoted between veterinarians, owners, and dog breeders. However, it should be kept in mind that the success of hip dysplasia control programs depends more on the knowledge of the breeding population than on the dog alone, and databases with reliable medical information are essential (25, 26).

## Conclusions

Pelvic tilting and DI measured using the Dis-UTAD showed the statistical reproducibility of the CertD measurements. The mean DI difference of 0.001 might be considered without clinical importance. The Dis-UTAD might be considered adequate to promote dog hip laxity.

## Data Availability Statement

All datasets generated for this study are included in the article/Supplementary Material.

## Ethics Statement

The animal study was reviewed and approved by Comissão de Ética da Universidade de Trás-os-Montes e Alto Douro (CE-UTAD). Written informed consent was obtained from the owners for the participation of their animals in this study.

## Author Contributions

AS contributed to the acquisition of data and drafting of the manuscript. SA-P contributed to the critical revision of the manuscript. JM contributed to the acquisition of data and critical revision of the manuscript. BC contributed to the concept/design and critical revision of manuscript. MG contributed to the concept/design, acquisition of data, data analysis/interpretation, and drafting of the manuscript. All authors contributed to the article and approved the submitted version.

## Conflict of Interest

The authors declare that the research was conducted in the absence of any commercial or financial relationships that could be construed as a potential conflict of interest.
